# Comparison of Phenology Models for Predicting the Onset of Growing Season over the Northern Hemisphere

**DOI:** 10.1371/journal.pone.0109544

**Published:** 2014-10-03

**Authors:** Yang Fu, Haicheng Zhang, Wenjie Dong, Wenping Yuan

**Affiliations:** 1 State Key Laboratory of Earth Surface Processes and Resource Ecology, Beijing Normal University, Beijing, China; 2 State Key Laboratory of Cryospheric Sciences, Cold and Arid Regions Environmental and Engineering Research Institute, Chinese Academy of Sciences, Lanzhou, Gansu, China; DOE Pacific Northwest National Laboratory, United States of America

## Abstract

Vegetation phenology models are important for examining the impact of climate change on the length of the growing season and carbon cycles in terrestrial ecosystems. However, large uncertainties in present phenology models make accurate assessment of the beginning of the growing season (BGS) a challenge. In this study, based on the satellite-based phenology product (i.e. the V005 MODIS Land Cover Dynamics (MCD12Q2) product), we calibrated four phenology models, compared their relative strength to predict vegetation phenology; and assessed the spatial pattern and interannual variability of BGS in the Northern Hemisphere. The results indicated that parameter calibration significantly influences the models' accuracy. All models showed good performance in cool regions but poor performance in warm regions. On average, they explained about 67% (the Growing Degree Day model), 79% (the Biome-BGC phenology model), 73% (the Number of Growing Days model) and 68% (the Number of Chilling Days-Growing Degree Day model) of the BGS variations over the Northern Hemisphere. There were substantial differences in BGS simulations among the four phenology models. Overall, the Biome-BGC phenology model performed best in predicting the BGS, and showed low biases in most boreal and cool regions. Compared with the other three models, the two-phase phenology model (NCD-GDD) showed the lowest correlation and largest biases with the MODIS phenology product, although it could catch the interannual variations well for some vegetation types. Our study highlights the need for further improvements by integrating the effects of water availability, especially for plants growing in low latitudes, and the physiological adaptation of plants into phenology models.

## Introduction

Phenology refers to the timing of recurring biological cycles, and is considered a sensitive indicator of climate change [1]–[3]. In particular, as research interest in global change increases, determining the beginning of the growing season (BGS) of land vegetation has become an important research subject [4]. Previous studies revealed that plant activity is more sensitive to climatic changes in spring than other seasons; and changes in the BGS would strongly impact the seasonal energy balance and net carbon dioxide (CO2) flux of terrestrial ecosystems [5], [6].

Large uncertainties, however, in present phenology models make accurate assessment of BGS a challenge. Two classes of process-based models have been developed for simulating the spring phenological phases. Models belonging to the first class, the ‘one-phase’ models, are the simplest and have been used in agronomy since the 18^th^ century [7]. This kind of model implicitly assumes that bud dormancy is fully released after a fixed sum of accumulated temperatures has been reached. The second class of models, the ‘two-phase’ models, considers the breaking of two dormancy phases [8]. The first phase is a period when buds remain dormant due to plant endogenous factors, and the second phase is a period when buds remain dormant due to unfavorable environmental factors [9]. Many studies have described the breaking of the first phase and overcoming the second phase in terms of chill accumulation to break the first phase followed by a period of forcing temperature to overcome the second phase [10], [11]. The two-phase models are of more recent development, and are conceptually based on experimental studies which highlighted that chilling was the major factor responsible for dormancy release [12]–[15].

Many phenology observations have focused on cultivated rather than natural plants [16], [17]. Geographically, most of the observations were conducted in North America and Europe [18]–[20]. Due to the limited availability of phenological observation data on a large scale, most phenology models are calibrated at local scales [21] and thus are unlikely to accurately predict BGS across different vegetation types. These phenology models might underestimate or overestimate the BGS when applied to a regional or global scale [22]. For example, a comparison of phenology models in 14 terrestrial biosphere models indicated that almost all models failed to track the phenology, and most predicted an earlier BGS, overestimating the gross ecosystem photosynthesis by 20% [23].

Remote sensing data from satellites provide broad coverage of useful information on vegetation phenology for diverse ecosystems at various scales, and help to calibrate the phenology models [24]–[28]. For example, Yang et al. [22] parameterized three budburst models in New England using 11 years of remotely sensed phenology and climate data. Nowadays, remote sensing-based phenology has been significantly improved with the Moderate Resolution Imaging Spectroradiometer (MODIS) on board the Terra and Aqua satellites [29]. Since 2009, the latest version of the MODIS Land Cover Dynamics Product (MCD12Q2) has been available [30], which provides valuable phenology data for the present study.

Based on the global satellite-based phenological observations, the primary objectives of this study are to (1) calibrate four phenology models; (2) compare the relative strengths of four phenology models; and (3) assess the spatial pattern and interannual variability of BGS in the Northern Hemisphere.

## Data and Methods

### 1. Satellite and meteorological data

The V005 MODIS Land Cover Dynamics (MCD12Q2) product (informally called the MODIS Global Vegetation Phenology product) was used to estimate the vegetation phenology of the study area. It identifies the vegetation growth, maturity, and senescence that mark seasonal cycles at global scales with a 500 ×500 m spatial resolution and is available from 2001 to 2010 [30]. This product is produced each year from the 8-day vegetation index EVI (Enhanced Vegetation Index) calculated from the NBAR reflectance (Nadir Bidirectional Reflectance Distribution Function-Adjusted Reflectance). More complete details regarding algorithm implementation are provided in Zhang et al. [29] and Ganguly et al. [30].

**Figure 1:**
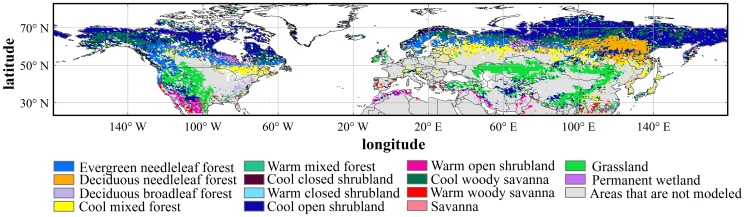
Vegetation distribution map of the Northern Hemisphere retrieved from the V005 MODIS Land Cover Type Product (MCD12Q1). Grey areas are either excluded vegetation types like croplands, or areas with no seasonal cycle detectable by satellite.

The V005 MODIS Land Cover Type Product (MCD12Q1) was used to identify land cover properties. It provides data characterizing five global land cover classification systems at annual time steps and 500 ×500 m spatial resolution for 2001-present. In this study, we chose the International Geosphere Biosphere Programme (IGBP) classification scheme, which includes 11 natural vegetation classes, three developed and mosaicked land classes, and three non-vegetated land classes. We excluded the evergreen broadleaf forest from our analysis as it has little or no leaf seasonal cycle. We also excluded croplands and crop/natural vegetation mosaics because human management practices strongly impact their phenology (e.g. irrigation, fertilization). In the classification of IGBP, a single vegetation type may exist in both subtropical and boreal regions (e.g. woody savannah in Figure 1). As plants in different regions require markedly different quantities of heat, it is necessary to subdivide vegetation types according to the climatic conditions in order to get the optimal model parameters. Therefore, we subdivided four vegetation types which are distributed across a wide range of latitudes, based on the climate criteria of Botta et al. [31]. Three meteorological variables were used to identify the vegetation types, including the annual mean of daily temperature (T_mean_), the minimum daily temperature of the year (T_c_) and the difference between annual maximum (T_w_) and minimum daily temperatures (ΔT = T_w_– T_c_) (Table 1).

**Table 1:** Climate criteria used to subdivide the four vegetation types which are distributed across a wide range of latitudes.

Vegetation type	Subdivision	Climate criteria
Mixed forest	Cool mixed forest	T_C_<0°C
	Warm mixed forest	T_C_≥0°C
Closed shrub	Cool closed shrub	T_C_<0°C
	Warm closed shrub	T_C_≥0°C
Open shrub	Cool open shrub	△T>20°C or T_C_<5°C
	Warm open shrub	△T≤20°C and T_C_≥5°C
Woody savanna	Cool woody savanna	T_C_<0°C
	Warm woody savanna	T_C_≥0°C

The climate criteria is gained from Botta et al. [31]. T_C_ and ΔT are respectively the minimum daily temperature of the year (T_C_) and the difference between annual maximum (T_W_) and minimum daily temperatures (ΔT =  T_W_–T_C_).

Daily meteorological data, including temperature and precipitation, were derived from the MERRA (Modern Era Retrospective-Analysis for Research and Applications) archive for 2001–2010. MERRA is a NASA reanalysis for the satellite era using a major new version of the Goddard Earth Observing System Data Assimilation System Version 5 (GEOS-5) [32]. MERRA uses data from all available surface weather observations globally every 3 hours. The GEOS-5 is used to interpolate and grid these point data on a short time sequence, and produces an estimate of climatic conditions for the world at 10 m above the land surface (i.e., approximating canopy height conditions). The resolution is 0.5° latitude by 0.67° longitude. The MERRA reanalysis dataset has been validated carefully at the global scale using surface meteorological data sets to evaluate the uncertainty of various meteorological factors (i.e. temperature, radiation, humidity, precipitation and energy balance). Detailed information on the MERRA dataset is available at the website (http://gmao.gsfc.nasa.gov/research/merra).

### 2. Phenology Models

In this study, we compared three one-phase phenological models for the beginning of growing season (BGS) including the Growing Degree Day model (GDD), the Biome-BGC phenology model (BBGC) and the Number of Growing Days model (NGD), and a two-phase phenological model (the Number of Chilling Days-Growing Degree Day model (NCD-GDD)) over the Northern Hemisphere (Figure 1). We did not include the Southern Hemisphere and tropical regions because of the poor performance of the V005 MODIS Land Cover Dynamics (MCD12Q2) product over these regions [25].

The GDD model is a classical one-phase phenological model, and has been used to predict the timing of BGS in spring by a function of accumulated temperature [33], [34]. After a starting date t_0_ (usually January 1st), mean air temperature above a degree-day base temperature (T_th_GDD_) is accumulated until a critical value (GDD_c_) is exceeded; at that time (t_1_) the prescribed growing season starts. The model can be described as follows:
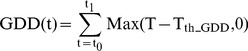
(1)

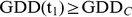
(2)


The BBGC model is integrated into the Biome-BGC (BioGeochemical Cycles) terrestrial ecosystem process model, described in White et al. [21]. The BBGC model divides vegetation phenology into two types: woody plants (i.e. trees and brush) and grasses [35]. For deciduous woody plants, the growing season begins when the running sum of the daily average soil temperatures (when the average soil temperature is above a degree-day base soil temperature (T_th_BBGC_)) is above a critical value defined by:
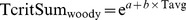
(3)


where T_avg_ is the mean daily average temperature; a and b are empirical coefficients. Moreover, the model specifies that the day length must be longer than 39300 seconds for leaf out to occur.

For grasses, the BGS is controlled by both temperature and water availability. When both of the accumulated soil temperatures and the accumulated precipitation values are larger than or equal to the critical values, the growing season begins. The critical accumulated soil temperature value (TcritSum_grass_) and the critical accumulated precipitation value (PrcpCritSum_grass_) for grasses are defined as:
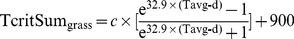
(4)

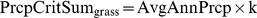
(5)


where AvgAnnPrcp is the annual mean precipitation; c is an empirical coefficient; d is an underdetermined soil temperature threshold which determines warm grasslands or cool grasslands; k is a proportion of the average annual precipitation. The actual leaf onset day is 15 days prior to this calculated date to estimate the start of the growing season. Soil temperature is assumed to be the 11 day running average of daily average temperature [36]. Detailed information on the BBGC model is available at the website (http://www.ntsg.umt.edu/project/biome-bgc).

The NGD model, proposed by Botta et al. [31], determines the BGS when the NGD, defined as the number of days with temperature above a base temperature (T_th_NGD_), exceeds a critical number of growing days (NGD_c_).

The NCD-GDD model is a two-phase model. Numerous experiments have confirmed that some plant species need to experience low temperatures to break physiological dormancy [37]. The NCD-GDD model defines the chilling days as the days with daily mean air temperature below a chill day base temperature threshold (T_th_NCD_). More chilling days can reduce the demand of plants for accumulated temperature [38]. The NCD-GDD model initiates bud burst if a certain relationship between the number of chilling days (NCD) since the leaves are lost, and the growing degree-days (GDD) since midwinter, is fulfilled, using the following empirical negative exponential law:
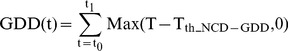
(6)

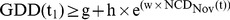
(7)


where T_th_NCD-GDD_ is the degree-day base temperature; g, h and w are empirical coefficients. We used the method of Murray et al. [12], starting summation from fixed dates: November 1^st^ for the number of chilling days (NCD_Nov_) to cover the major part of the dormant period, and January 1^st^ for GDD [31].

### 3. Parameter Inversion

In each vegetation type, we randomly selected one half of the pixels to calibrate model parameters, and validated the models at the other half pixels. The nonlinear regression procedure (Proc NLIN) in the Statistical Analysis System (SAS, SAS Institute Inc., Cary, NC, USA) was applied to optimize the parameter values of the four phenology models. We used the Newton method to train the data and got the optimal model parameters when the error sum of squares was minimized. The other options were set as the default. The details of the calibrated parameter values of the four phenology models are found in Table 2.

**Table 2:** Parameter values: the degree-day base temperature (T_th_GDD_) is estimated for model GDD (Eq. (1)); the critical value of growing degree days (GDD_C_) is estimated for model GDD (Eq. (2)); the degree-day base temperature (T_th_BBGC_) is estimated for model BBGC; empirical coefficients (a and b) are estimated for model BBGC (Eq. (3)); the empirical coefficient (c) is estimated for model BBGC (Eq. (4)); the underdetermined soil temperature threshold (d) determining warm grasslands or cool grasslands is estimated for model BBGC (Eq. (4)); the proportion (k) of the average annual precipitation is estimated for model BBGC (Eq. (5)); the base temperature (T_th_NGD_) and the critical number of growing days (NGD_C_) are estimated for model NGD; the degree-day base temperature (T_th_NCD-GDD_) is estimated for model NCD-GDD (Eq. (6)); the chill day base temperature (T_th_NCD_), and empirical coefficients (g, h, and w) are estimated for model NCD-GDD (Eq. (7)).

Biome	GDD		BBGC						NGD		NCD-GDD				
	T_th_GDD_ (°C)	GDD_C_ (°C*days)	T_th_BBGC_ (°C)	a	b	c	d	k	T_th_NGD_ (°C)	NGD_C_ (days)	T_th_NCD_ (°C)	T_th_NCD-GDD_ (°C)	g	h	w
Evergreen needleleaf forest	6	50	0	4.755	0.117	-	-	-	9	5	0	3	−300	400	−0.1
Deciduous needleleaf forest	2	52	0	4.755	0.117	-	-	-	5	6	0	−1	−300	400	−0.1
Deciduous broadleaf forest	−5	591	−5	5.505	0.085	-	-	-	6	21	0	−5	−100	700	−0.1
Cool mixed forest	5	52	0	4.63	0.101	-	-	-	8	6	−5	3	−300	400	−0.1
Warm mixed forest	7	236	−5	6.005	0.057	-	-	-	5	59	−5	5	−300	600	−0.1
Cool closed shrub	5	50	2	4.13	0.109	-	-	-	8	5	−5	3	−300	400	−0.1
Warm closed shrub	7	308	−5	6.63	0.041	-	-	-	5	77	−5	5	−300	700	−0.1
Cool open shrub	0	140	−3	5.505	0.057	-	-		5	11	−5	1	−300	400	−0.1
Warm open shrub	7	350	−5	6.005	0.069	-	-		10	41	−2	7	−300	600	−0.1
Cool woody savanna	3	58	0	-	-	15	119	0.05	7	6	−5	1	−300	400	−0.1
Warm woody savanna	7	296	−5	-	-	5	119	0.06	5	58	−5	5	−300	600	−0.1
Savanna	1	126	−2	-	-	11	209	0.16	5	12	−5	5	−300	400	−0.1
Grassland	−5	448	−5	-	-	15	369	0.05	6	12	−5	5	−300	400	−0.1
Permanent wetland	−5	378	−1	5.005	0.101	-	-	-	−4	47	−5	1	−300	400	−0.1

### 4. Model comparison

We made two comparisons in this study. First, we compared the original and calibrated models based on the start dates of phenology derived from the MODIS product for various of biomes. Second, we compared the start dates of phenology from calibrated models and the MODIS product over the northern hemisphere biomes. The performance of the parameterized and original phenology models is assessed by comparison with the results of the MODIS Land Cover Dynamics dataset. For the phenological data, all dates were transformed to days of the year (DOY) for convenience of data analysis.

## Results

**Figure 2:**
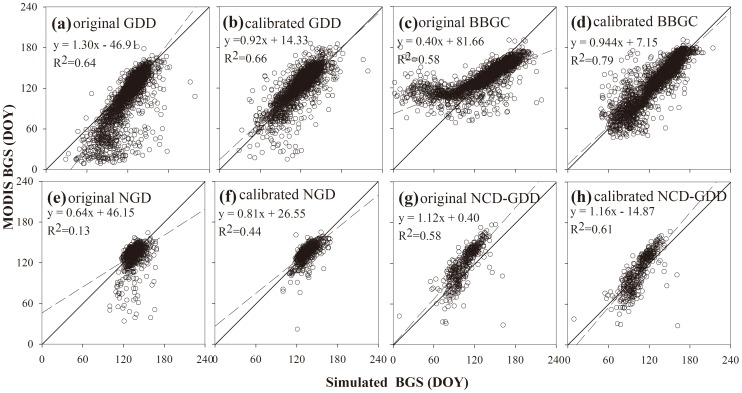
The correlations between MODIS BGS and simulated BGS. (a) and (b) show BGS simulations derived from GDD models with the original parameter values in IBIS model and calibrated parameters respectively; (c) and (d) show BGS simulations of deciduous forest and grassland derived from BBGC models with the original parameter values in Biome-BGC model and calibrated parameters respectively; (e) and (f) show BGS simulations of deciduous needle leaf forest derived from NGD models with the original parameter values in ORCHIDEE model and calibrated parameters respectively; (g) and (h) show BGS simulations of deciduous broadleaf forest derived from NCD-GDD models with the original parameter values in ORCHIDEE model and calibrated parameters respectively. The solid line is the 1∶1 line and the short dashed lines are regression lines.

Model parameterization significantly improved performance of the four models. We calibrated and examined the four phenology models used in the global dynamic vegetation models based on satellite phenology observations over the Northern Hemisphere. The performance of the calibrated phenology models was better than that of the original models. For example, in the Integrated Biosphere Simulator (IBIS) model, the GDD phenology model is used to estimate the BGS for winter-deciduous forest, grassland and shrub [39]. For winter-deciduous forest, the original parameters values of T_th_GDD_ and GDD_C_ were set as 0°C and 100 degree-days, respectively. For grassland and shrub, the original parameters values of T_th_GDD_ and GDD_C_ were set as 5°C and 150 degree-days, respectively. The results of the calibrated simulation more accurately predicted the BGS, giving higher R^2^ (Figure 2a and b). Similar results were also found in the respective phenology modules of the Biome-BGC and the Organising Carbon and Hydrology in Dynamic Ecosystems (ORCHIDEE) models (Figure 2). In the ORCHIDEE model, the NGD model and the NCD-GDD model are used in the deciduous needle leaf forest and deciduous broadleaf forest, respectively. Therefore, we compared the BGS simulations at these two vegetation types with the original parameter values and calibrated parameters in the ORCHIDEE model, respectively.

**Figure 3:**
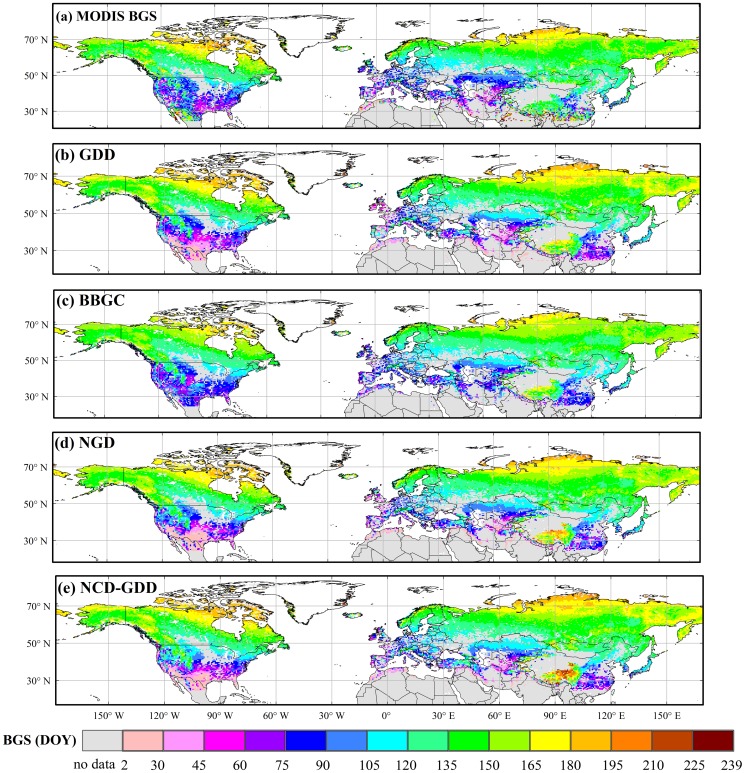
Spatial pattern of mean dates for the beginning of growing season (BGS) in the Northern Hemisphere during 2001–2010. (a) The start dates derived from the MODIS product; (b)–(e) indicate the simulated start dates of the four phenology models.

**Figure 4:**
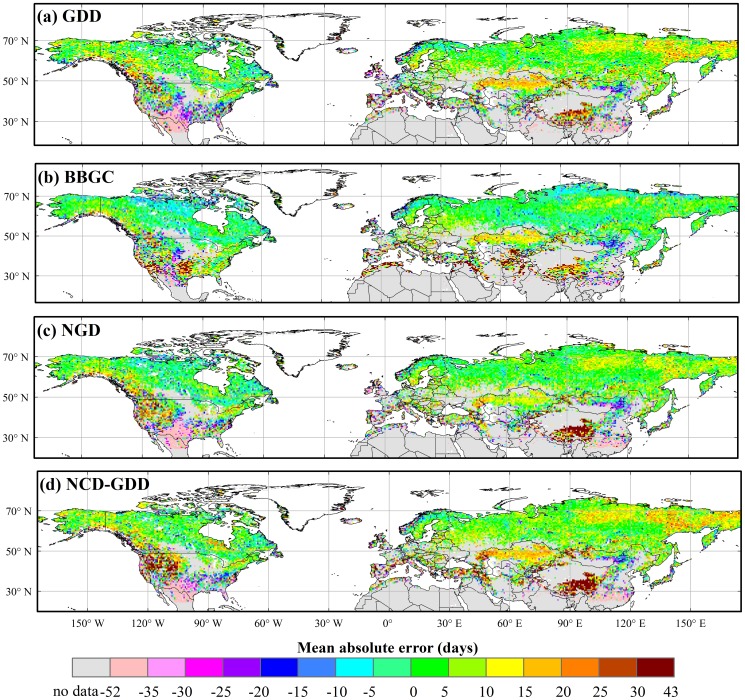
Spatial pattern of the mean absolute error (R_A_) of the BGS simulations from four phenology models in the Northern Hemisphere. The mean absolute error (R_A_) values are derived from the comparison of the MODIS vegetation product results with those of the parameterized models.

**Figure 5:**
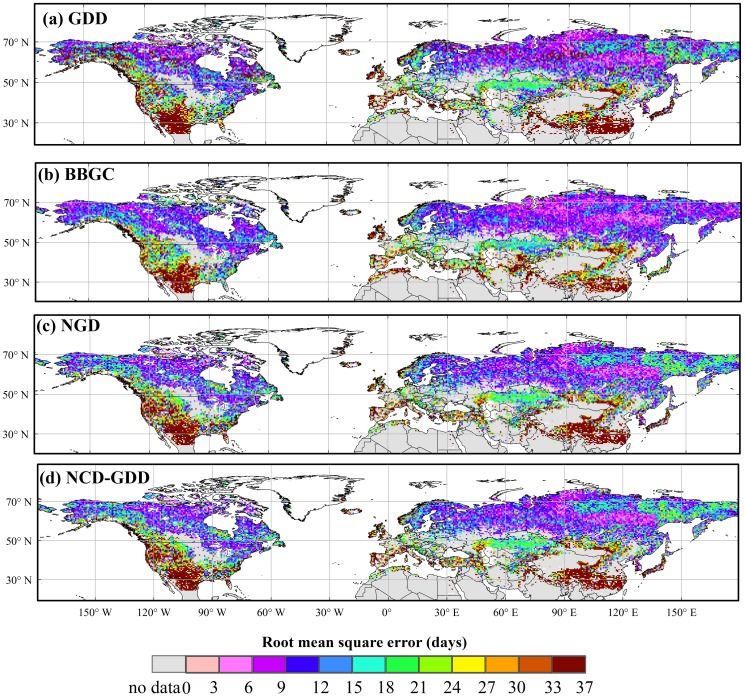
Spatial pattern of the root mean square error (RMSE) of the BGS simulations from four phenology models in the Northern Hemisphere. The RMSE values are derived from the comparison of the MODIS vegetation product results with those of the parameterized models.

**Figure 6:**
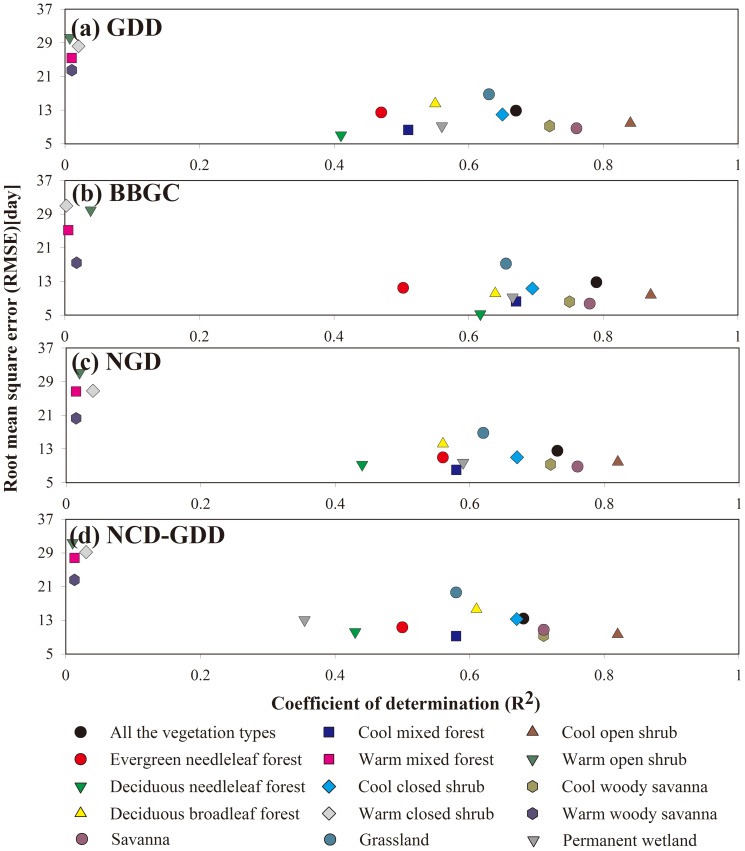
Coefficient of determination (R^2^) and root mean square error (RMSE) for four phenology models at various vegetation types over the Northern Hemisphere. The R^2^ and RMSE values are derived from the comparison of the MODIS vegetation product results with those of the parameterized models.

All of the four calibrated phenology models simulated similar spatial patterns of the BGS, which agreed very well with those of the MODIS BGS (Figure 3). A late BGS was found in the boreal and cool regions, intermediate BGS at temperate regions and early BGS in warm regions. In terms of the spatial patterns of the mean absolute error (R_A_) and the root mean square error (RMSE), the four models showed good performance in most vegetation types (Figure 4 and Figure 5). The results showed low RMSE and R_A_ of the BBGC simulations in most boreal and cool regions. The average RMSE value in the whole study area was 16±15days (mean±1SD). The GDD, NGD and NCD-GDD models showed higher RMSE, with average values of 20±19 days (mean±1SD), 19±18 days (mean±1SD) and 22±20 days (mean±1SD), respectively (Figure 5). In contrast, all of the four models showed poor performance at the four warm vegetation types (i.e. warm mixed forest, warm closed shrub, warm open shrub and warm woody savanna). The coefficient of determination (R^2^) in warm mixed forest, warm closed shrub, warm open shrub and warm woody savanna regions were close to zero, and the average RMSE was in the range of 17–31 days (Figure 5 and Figure 6). In addition, all of the four phenology models predicted a later BGS in the grassland areas of the Qinghai–Tibet Plateau.

**Figure 7:**
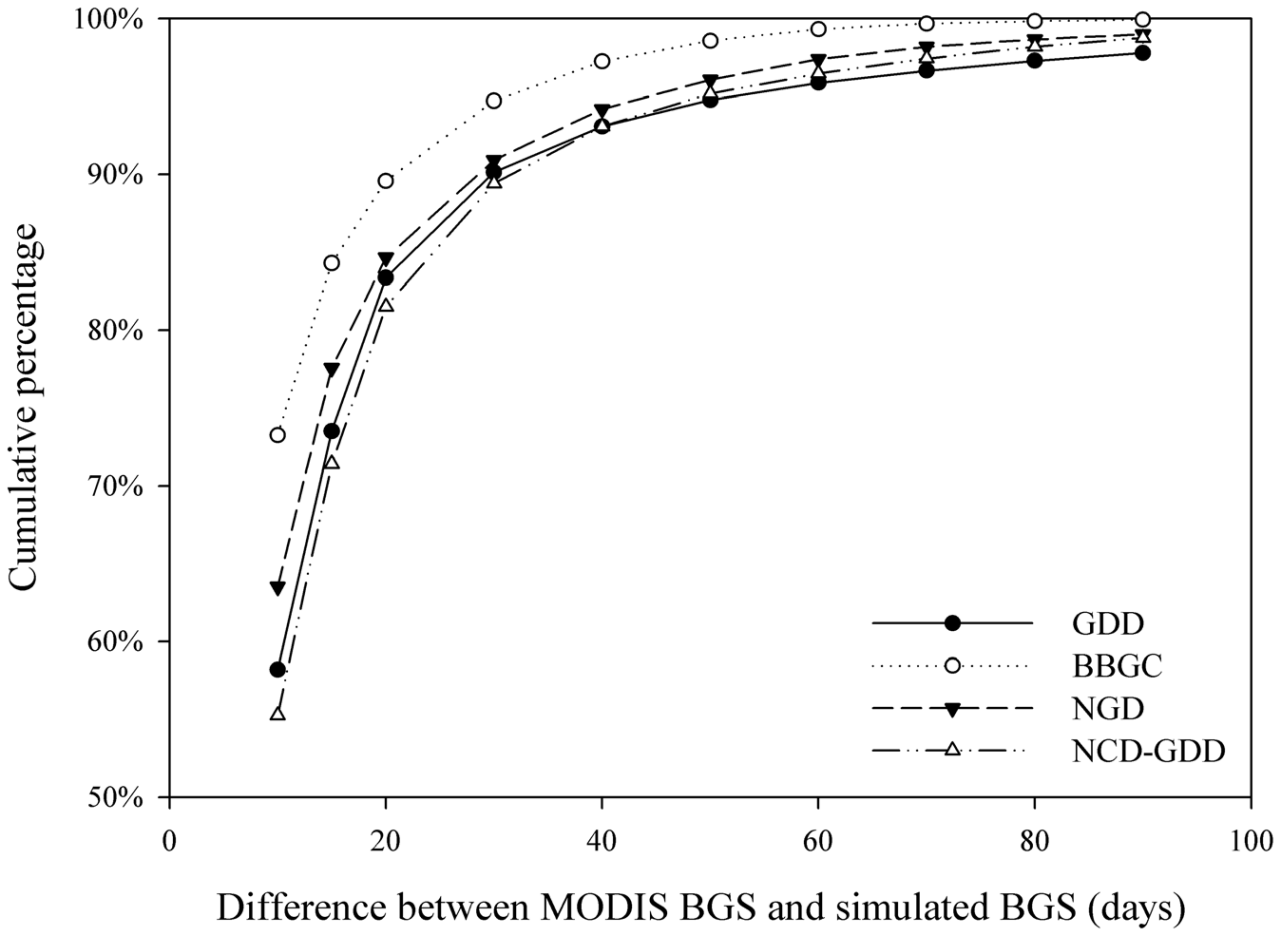
Cumulative percentage of absolute differences between MODIS BGS and simulated BGS from four models.

The results showed large differences in simulated BGS among the four phenology models (Figure 6). On average, they explained about 67% (GDD), 79% (BBGC), 73% (NGD) and 68% (NCD-GDD) of the BGS variations over the Northern Hemisphere (Figure 6). According to the average R^2^ and RMSE, the BBGC model showed the best performance with the highest R^2^ (0.50–0.87) for the 9 vegetation types and lowest RMSE (5–11 days) (Figure 6b). The GDD and NGD models showed relatively similar performance in almost all vegetation types. In contrast, the NCD-GDD model showed a slightly lower R^2^ (0.35–0.82) compared with the other models in most vegetation types, with RMSE ranging from 6 to16 days (Figure 6d). The cumulated frequencies of absolute difference between simulations and the MODIS BGS further demonstrated different simulation accuracy (Figure 7). On the whole, the best estimate was the BBGC model, which reproduced the timing of BGS for 73.2% of the pixels in the study areas within 10 days of the MODIS BGS, and for 84.3% within 15 days (Figure 7). Similarly, the NGD model reproduced the timing of BGS for 63.5% of the pixels within 10 days of the MODIS BGS, and for 77.6% within 15 days. The GDD and NCD-GDD models performed slightly worse and reproduced the timing of BGS for 58.2 and 55.3% within 10 days of the MODIS BGS, and for 73.5 and 71.4% within 15 days, respectively.

**Figure 8:**
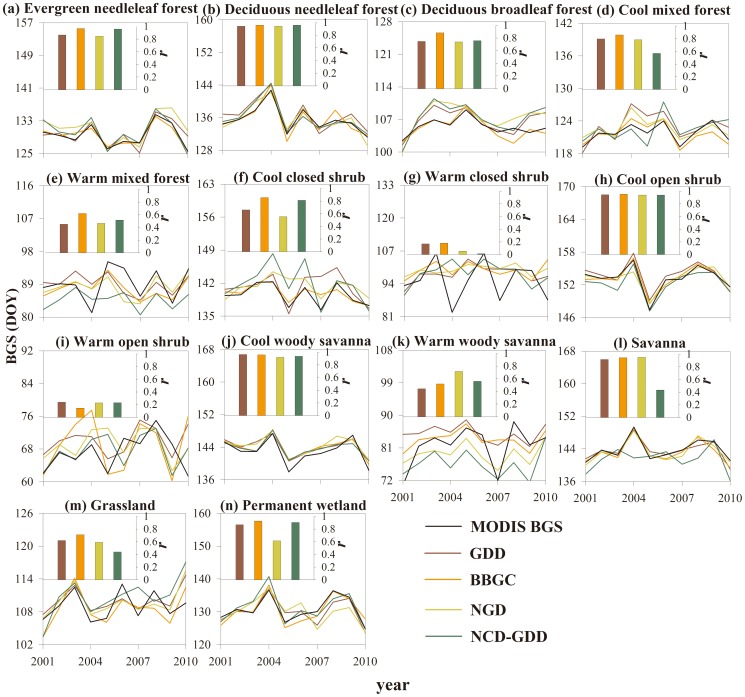
Interannual variability of the start dates of growing season from MODIS product and four phenology models from 2001 to 2010. The inset panels show the correlation coefficient (*r*) between BGS simulations of the four phenology models with MODIS BGS.

The magnitude and long-term change trends of the date of BGS differed significantly among the four phenology models (Figure 8). The Pearson's correlation coefficient (*r*) was used to quantify the performance of the four models in different vegetation types (Figure 8). The BBGC model had the highest *r* for almost all vegetation types, with the average value of 0.75. The GDD and NGD models showed relatively similar performance of *r* between simulations and the MODIS BGS, with the average values of 0.69 and 0.67, respectively. The NCD-GDD model had the lowest *r*, with an average value of 0.64.

## Discussion

Vegetation phenology plays an important role in the functioning of the earth system as it steers the exchanges of carbon, water and energy between vegetation and the atmosphere [40], [41]. The changes of phenology periods may significantly impact the ecosystem and climate system [42], [43]. For example, an advanced spring may enhance carbon sequestration and affect species interactions, and then alter the structure and function of ecosystems [44], [45]. Therefore, the phenology module is one of the most important components of dynamic vegetation models and earth system models [46], [47].

This study examined four phenology models, which have been widely integrated into various global dynamic vegetation models [39], [48]–[50]. The major parameters of the original phenology models have not been carefully estimated or only calibrated over local scales [21], [22], [51]. For example, White et al. [21] used satellite data to calibrate a phenology model which was integrated into the Biome-BGC model, but this was only conducted at the North American not the global scale. Therefore, large biases in predicting the BGS exist among phenology modules, resulting in poor performance of these dynamic vegetation models [23]. This study calibrated and examined the four phenology models used in the global dynamic vegetation models based on satellite phenology observations over the Northern Hemisphere. When the parameters were calibrated, the performance of the calibrated phenology models was better than that of the original models.

The four temperature-driven phenology models showed poor performance for vegetation in low latitude areas (i.e. warm mixed forest, warm closed shrub, warm open shrub and warm woody savanna). Experimental evidence indicates that plant growth may be largely controlled by precipitation and drought stress for these plant species [52]. For instance, Bernal et al. [53] studied the phenology of a Mediterranean shrub, *Erica multiflora*, and found that its growth was mainly driven by precipitation. Moreover, other studies also indicated that the plant phenology in low latitude areas was responsive to rainfall and water availability (e.g. Peñuelas et al. [54]). However, many phenological models for the low-latitude plant species are found to be solely driven by temperature [21], [39], [50], [55]. Thus, it is important to integrate water availability in plant phenology models when simulating the BGS of low latitudes.

Overall, the BBGC model showed better model performance than the other one-phase models (GDD and NGD). To account for this, we attributed two reasons. First, the BBGC model uses the mean annual temperature to determine the threshold of growing degree-days [21]. Under the local environmental conditions, vegetation phenology is the optimization of the plant activity and reproduction resulting from natural selection [56]. Plant species have adapted their temperature requirements to their local environment [10], [57], [58]. The BBGC model is essential in order to integrate the physiological adaptation of plants to the local temperature into the models and improve model performance at the global scale. Second, the BBGC model added the precipitation component to the start of the growing season calculation for grass biomes.

Moreover, the two-phase phenology model (NCD-GDD) did not perform better than the one-phase models in most vegetation types. Although it could simulate the interannual variations well for some vegetation types, it showed larger biases in the whole North Hemisphere. This result is consistent with other studies [11], [59]. For example, Yuan et al. [11] analyzed the phenological characteristics of two dominant grass species for one-phase and two-phase models and also found better performance of the one-phase model. Leinonen and Kramer [33] also found that chilling was not important for good performance of models and proposed two explanations: first, with a boreal climate, winter temperatures are so low that the chilling requirement will always be fulfilled; second, the chilling requirement is observed to be lower for northern tree species and provenances compared to southern ones, i.e. relatively short exposure to low temperature is sufficient to break bud dormancy.

Our study was based on the MODIS Land Cover Dynamics (MCD12Q2) product, and the uncertainties from this product have a certain impact on the simulated results of phenology models. For example, a previous study compared the BGS derived from MODIS product with field measurements of forest canopy phenology at Harvard Forest for 2001–2006 and found differences of 1–17 days in each of the six years [30]. In addition, the MODIS BGS showed large uncertainties in the tropics [25]. Overall, ongoing efforts focusing on improving the precision of the phenology product are needed to improve phenology models.

## Summary

In the present study, we calibrated four temperature-driven phenology models and compared their performances in the Northern Hemisphere. Although all of the four models indicated similar spatial patterns of the BGS, there were substantial differences among the models. The four models explained 67–79% of the variability in BGS. The BBGC model showed better performance than the other models. Conversely, the NCD-GDD model showed larger biases compared with the other three models in the whole North Hemisphere, although it could simulate the interannual variations well for some vegetation types. Moreover, all models showed good performance for most types in cool regions but poor performance in warm regions. Our study showed that it is necessary to integrate the effects of water availability into phenology models, especially for plants growing in low latitudes. Moreover, the thresholds used in phenology models to determine the BGS should be location dependent rather than a constant, as plants growing in different places show different physiological adaptabilities to environments (such as cold tolerance and drought tolerance).
